# Development and evaluation of an integrated multispecialty clinic for people with multiple long-term conditions

**DOI:** 10.1016/j.fhj.2025.100235

**Published:** 2025-03-04

**Authors:** Michael E Reschen, Jennifer J Rayner, Gaya Thanabalasingham, Alistair Lumb, Michael Matheou, Sophie McGlen, Nayia Petousi, Luke Solomons, Rustam D Rea, Christopher A O'Callaghan

**Affiliations:** a Department of Acute General Medicine, John Radcliffe Hospital, Oxford University Hospitals NHS Foundation Trust, Oxford, UK; b Department of Cardiology, John Radcliffe Hospital, Oxford University Hospitals NHS Foundation Trust, Oxford, UK; Division of Cardiovascular Medicine, Radcliffe Department of Medicine, University of Oxford, Oxford, UK; c Oxford Centre for Diabetes, Endocrinology and Metabolism, Churchill Hospital, University of Oxford, Oxford, UK; d Oxford Centre for Diabetes Endocrinology and Metabolism and NIHR Oxford Biomedical Research Centre, Oxford University Hospitals NHS Foundation Trust, Oxford, UK; e NIHR Oxford Biomedical Research Centre and Respiratory Medicine Unit, Nuffield Department of Medicine, University of Oxford, Oxford, UK; f Department of Pyschological Medicine, John Radcliffe Hospital, Oxford University Hospitals NHS Foundation Trust, Oxford, UK; g Nuffield Department of Medicine, Centre for Human Genetics, University of Oxford, Oxford, UK

## Abstract

**Introduction:**

The number of people with multiple long-term conditions (MLTC) is increasing. People with MLTC experience fragmentation of care due to single-disease-orientated healthcare organisation and have increased morbidity and mortality. We developed an innovative clinic model whereby people with MLTC are assessed by a team of specialists together in one appointment to form a consensus management plan in real time. We report the outcomes together with patient and clinician feedback.

**Methods:**

A multispecialty clinic was established to assess adults referred from the community or secondary care with MLTC. Patients were seen together by three or more relevant specialists and a coordinated plan was developed. To evaluate the clinic, we collected patient outcomes and obtained feedback from patients and clinicians in the clinic and from primary care clinicians.

**Results:**

Twenty seven patients were assessed in the multispecialty clinic with a mean age of 64.6 years; 89% had diabetes, 70.4% hypertension, 63% CKD stages 3–5, and 51.9% had heart failure. Patients were taking a mean of 10 medications. Referrals were from primary care (51.9%) and secondary care, with the commonest reason being fluid overload (29.6%). On average, 1.7 medication changes were made per patient. Compared to the 6-month period before the clinic, in the 6 months after the clinic there was a significant reduction in interactions of patients with the acute hospital services (emergency department, same-day emergency care unit and acute inpatient medicine service). In a survey of 11 patients, all reported high satisfaction with the novel clinic format. A survey of seven clinicians, including a pharmacist and trainee doctor, demonstrated positive experiences of the clinic, confidence in the clinical decision making and enhanced learning. Primary care physicians also appreciated the coordinated plan across several specialties.

**Discussion:**

In our pilot multispecialty clinic, people with MLTC were able to develop a real-time consensus plan with a group of specialists. Our approach was associated with fewer unscheduled healthcare interactions after the clinic. Our after-clinic survey showed positive responses from patients and clinicians. Future studies could examine how such a service could be rolled out to a wider group of people effectively and efficiently.

## Highlights

• We developed a multispeciality clinic to streamline and defragment care for patients with multiple long-term conditions (MLTC).
• Patients met multiple specialists in one room together to develop a consensus plan.
• Analysis of data before and after the clinic showed a marked reduction in unscheduled acute hospital attendance after the clinic.
• Surveys of both patients and clinicians working in the clinic showed high levels of satisfaction, highlighting enhanced multidisciplinary working, and improved patient understanding.

## Introduction

Providing optimal care for people with multiple long-term conditions (MLTC) is increasingly recognised as a core challenge for modern healthcare systems, which developed largely around the management of individual diseases in isolation.^1^^,^^2^ An increasingly large proportion of the population have MLTC and may be served poorly by unrelated and uncoordinated encounters with single-disease or single-organ services that cannot readily consider their ongoing lived experience with multiple conditions.

MLTC is defined by the presence of two or more long-term health conditions and its prevalence is as high as one-third of adults.^3^ People with MLTC experience reduced quality of life, increased hospital admission rates, longer hospital stays and increased mortality – the latter being particularly evident during the COVID-19 pandemic.^4^, ^5^, ^6^, ^7^, ^8^

People with MLTC have increased healthcare costs.^9^^,^^10^ In a Danish study of over 3 million people, 23% had MLTC, but they accounted for 62% of healthcare expenditure.^11^ In the US Veterans Administration healthcare system, 5% of patients accounted for half of the overall healthcare spending and two-thirds of these patients had MLTC.^12^

The traditional focus of healthcare systems on single-disease problems has resulted in the development of specialty guidelines, the organisation of clinic services, the education and training of clinicians, the execution of clinical trials and many other aspects of practice that fail to factor in the more complex needs of people with MLTC and serve to perpetuate this failure.^13^ For people with MLTC and their clinicians, this can lead to a frustrating and wasteful fragmentation of care whereby they circulate through a variety of unconnected specialty clinics. Patients gather sometimes disparate recommendations with resultant confusion for them, for their primary care physicians and the specialists, about how to integrate these different opinions.^14^ There is strong evidence that people with MLTC feel that there is a lack of holistic care as well as a lack of communication and coordination between the different professional and specialty teams about their treatment.^15^ There are also economic and resource implications around multiple and potentially redundant interactions with different services.

Recognition of the problems around MLTC has led to calls for research on how care could be integrated to provide unified management plans,^2^ and ‘organisation of care’ was the top research priority in the NICE (National Institute for Health and Care Excellence) guidance on MLTC.^16^ Suggestions for how to improve care have included broadening postgraduate medical training programmes, generating guidelines that combine advice for conditions that cluster together, including people with MLTC in relevant clinical trials and reorganising services to integrate multiple specialties with each other and with primary care.^2^

Developing useful interventions may be challenging and very few have been reported. A Cochrane review found only 17 studies, few of which addressed those with the most common constellations of MLTC and few of which included specialty care.^17^ A virtual multidisciplinary review of patients in a heart failure clinic who had MLTC noted a reduction in clinic attendances and all-cause hospitalisations as well as cost savings.^18^ A Canadian study randomised patients in a renal clinic who had MLTC to a combined clinic or to standard care in multiple clinics and demonstrated a better patient experience and cost savings.^19^ Patients were selected based on renal clinic attendance rather than perceived need for a multi-specialty approach. A Danish study demonstrated the feasibility of arranging different clinic visits to be on the same day for patients attending several specialty clinics. This reduced blood tests by 29% and clinician feedback was positive.^20^ A volunteer task force in the USA has developed multispecialty practice recommendations for people with diabetes, chronic kidney disease and heart failure and metabolic conditions, but this is necessarily mainly a compendium of recommendations based on studies of single diseases.^21^ Overall, there is a lack of evidence about how best to organise and provide healthcare for people with MLTC.

To address the problem of fragmented care for people with MLTC, we developed a multispecialty clinic in which people meet with a team of specialists simultaneously and together generate a consensus management plan. To evaluate this clinic, we collected patient information and outcomes, and obtained feedback from patients, from clinicians in the clinic and from primary care clinicians.

## Methods

The clinic was established at a hospital in the UK and was available for adult patients of any age. Each clinic session was typically staffed by a lead general medical consultant, a nephrologist, cardiologist and diabetologist, and other specialties as required, including respiratory medicine and psychological medicine (consultant psychiatrist). Nursing and pharmacy support were also available as required. Resident doctors were encouraged to attend for training purposes. The clinics were held in a hospital setting. New patient appointments typically lasted around 45–60 min. People were referred to the clinic from multiple sources. People could be referred directly from primary care and links were established on a pilot basis with a large local practice. People could also be referred from specialty clinics and from the same-day emergency care medicine service. Several referrals came from the SCAN (Suspected CANcer) clinic – a clinic for rapid investigation of non-specific symptoms that could indicate cancer – and cross-referral to our clinic arose where cancer was excluded and MLTC were present.^22^ Impaired mobility was not an exclusion criterion and hospital transport and assistance with mobility were available as needed. Following an appointment, a letter to the GP was drafted by one clinician and circulated for approval or editing by the other specialists to ensure clarity and consensus in the documentation for both the patient and their GP. Letters were routinely sent to patients and, to the maximal extent possible, were written to be easily understood using non-technical language.

Clinical data were collected from the electronic patient record and anonymised for analysis. Feedback from clinicians was undertaken in person or over Microsoft Teams. Following their clinic appointment, patients were invited to provide feedback by phone using a semi-structured interview format, which included a mixture of yes/no/unsure, Likert scale and open follow-up questions (see Supplementary Information). Survey results were anonymised. This was an observational cohort study undertaken as a quality improvement project. The clinic project was registered formally on the Oxford University Hospitals NHS Foundation Trust QIP (quality improvement project) database using Ulysses software.

## Results

### Patient characteristics and outcomes

#### Demographics

We assessed 27 people in the clinic and their characteristics are included in Table 1.

**Table 1:** Clinical characteristics of patients attending the multispecialty clinic.

Characteristic	Number (%)
Number of patients	27
Mean age (range), years	64.6 (32–89)
Male	16
Female	11
Comorbidity	
Diabetes type 1	1 (3.7)
Diabetes type 2	23 (85.2)
Mean HbA1c mmol/mol with diabetes (HbA1c as percentage)	73.6 (8.7%)
Diabetes with HbA1c > 100 mmol/mol	5 (18.6)
Heart failure	14 (51.9)
CKD stage 3 or greater	17 (63.0)
eGFR < 30 mL/min/1.73 m^2^	8 (29.6)
Chronic respiratory disease	8 (29.6)
Ischaemic heart disease	14 (51.9)
Hypertension	19 (70.4)
Atrial fibrillation	5 (18.6)
Medications	
Number of medications (mean)	10
ACEi, ARB or Sacubutril/valsartan	16 (59.3)
SGLT2 inhibitor	3 (11.1)
Insulin	12 (44.4)

The mean age of those seen was 64.6 years (range 32–89). Patients had varying levels of social deprivation, with 63% living in areas associated with living environments below the national mean. The clinic was routinely staffed by consultants, with a generalist, a nephrologist, a cardiologist and a diabetologist and other specialties as required, including respiratory medicine and psychological medicine.

#### Referrals

The commonest source of referrals was primary care (14, 51.8%), followed by the same-day emergency care unit (6, 22.2%), a single-specialty clinic (4, 14.8%), the SCAN clinic (a service for rapid investigation of non-specific symptoms that could indicate cancer) (2, 7.4%) and the inpatient medicine service (1, 3.7%). People were often referred because of multiple current problems, the commonest being fluid overload (8, 29.6%), diabetes management (5, 18.5%), hypertension (4, 14.8%), shortness of breath (4, 14.8%) and weight loss (2, 7.4%). Other problems included anaemia, diagnostic uncertainty and pyrexia of unknown origin.

#### Comorbidity

Of the 27 people seen in the clinic, 24 (88.9%) had diabetes and the average HbA1c in these people was 73.6 mmol/mol (or 8.7%), with five people having an HbA1c greater than 100 mmol/mol (Table 1). Chronic kidney disease (CKD) was common, with 17 (63%) people having CKD stages G3–5 and eight having an eGFR below 30 mL/min/1.73 m^2^ (CKD stages G4–5). There was a range of cardiovascular problems; 14 people had ischaemic heart disease, 14 had heart failure, 19 had hypertension and five had atrial fibrillation. Other cardiovascular problems included mitral valve disease, tricuspid valve disease and stroke. Eight people had at least one chronic respiratory problem and this included asthma,^2^ chronic obstructive pulmonary disease (COPD)^4^ and interstitial lung disease;^1^ two people were using continuous positive airway pressure (CPAP) devices at night.

#### Medications

Overall, people were prescribed an average of 10 different regular medications. Sixteen people were taking an RAAS (renin–angiotensin–aldosterone system) inhibitor (either an ACE inhibitor or angiotensin receptor blocker or sacubutril/valsartan). Three people were taking an SGLT2 inhibitor and 12 people were taking insulin (Table 1). About 11 (41%) people were taking mental health-related medication.

### Clinic actions

#### Medication changes

The index clinic review resulted in a medication change for 24 patients (88.9%). There were 46 changes to medications at the index visits, representing a mean of 1.7 changes per person. Recommended medication changes largely pertained to cardiovascular disease, diabetes, chronic kidney disease, anaemia or vitamin D deficiency. The commonest individual change was to recommend initiation of an SGLT2 inhibitor.

#### Investigations requested

Seven people had a CT scan requested by the clinic and these included CT pulmonary angiography, CT chest/abdomen/pelvis, PET CT, and CT colonoscopy. Four people had an ultrasound scan, two had a cardiac nuclear perfusion scan and six people had either upper gastrointestinal endoscopy, colonoscopy or both.

#### Outpatient clinic usage

Following the clinic, 22 (81.5%) people were discharged to primary care with a plan for their follow-up and clarity about if or when re-referral might be appropriate. Five people were followed up on one subsequent occasion and one person was followed up twice.

In the 6 months prior to their multispecialty clinic appointment, people were seen in a mean of 2.5 different types of specialty clinic. The multispecialty clinic made only two referrals to new services – one to the vascular surgery team for serious peripheral vascular disease that was newly diagnosed in the clinic and one to the orthopaedic team for debilitating hip pain and restricted mobility due to osteoarthritis. Four patients with advanced and progressing CKD were offered further follow-up by the clinic nephrologist to plan their future options, given their high risk of end-stage kidney disease.

Two patients had new and appropriate clinic referrals made by primary care in the 6 months after the clinic. One patient had acute referrals to the TIA clinic and the rapid-access chest pain clinic, and another had a referral to the spinal clinic.

### Change in use of acute services

We assessed the usage of acute services in the 6 months before and after each patient’s first clinic visit (Table 2). The acute services included our hospital emergency department, ambulatory unit (same-day emergency care – SDEC) and inpatient medical service. In some cases, these occurred on the same day of attendance. For example, someone could attend the emergency department, then be referred to the SDEC unit and then be admitted under the acute general medicine inpatient service. SDEC attendances were either planned or unplanned, but it is important to recognise that any planned reviews would only arise as a consequence of a recent unplanned attendance. As Table 2 shows, there were highly significant reductions in acute service usage. Overall attendances with the acute services fell by 53% (*p* = 0.01). Attendances to the SDEC fell by 70.3% (*p* = 0.01). The number of days on which an interaction took place with an acute service fell by 67.8% (*p* = 0.03).

**Table 2:** Service attendances of patients attending the multispecialty clinic.

	Before clinic	After clinic	% Change	*p* value
ED visits (number of days)	15	13	−13.3%	0.42
SDEC unplanned visits (number of days)	16	7	−56.3%	0.05
SDEC planned visits (number of days)	21	4	−81%	0.02
SDEC total visits (number of days)	37	11	−70.3%	0.01
Number of AGM inpatient nights	116	31	−73.3%	0.10
All acute service interactions (inpatient admissions only count as one interaction)	66	31	−53%	0.01
Days of interactions with all services (includes each day of inpatient stays)	171	55	−67.8%	0.03

Time periods represent the 6-month periods before and after the clinic appointment. ED, emergency department; SDEC, same-day emergency care. *p* values are calculated using the Wilcoxon rank sum test, paired for each person.

### Patient survey

We sought feedback from 11 consecutive patients seen in the clinic. We focused on aspects of the clinic that might differ from single-specialty clinics, which all patients had experience of attending. All 11 patients reported that the clinic experience was different from that in other clinics they had experienced. In terms of satisfaction, nine reported being ‘very satisfied’ overall and two being ‘satisfied’ overall with the clinic. All felt that the correct mix of specialists were present. Eight patients reported that their medical problems were all being addressed at once ‘very much’ and three reported ‘much’. When asked to what degree they felt listened to, all reported ‘very much’. All felt that there was enough time available during the appointment.

All agreed that clinicians communicated ‘very well’ with each other in clinic. Five patients reported that the extent to which the clinic helped them understand their condition better was ‘very much’ and six reported ‘somewhat’. Asked about potential options for the future set-up of the clinic, seven patients said that they preferred a face-to-face appointment and six patients said they would not mind if their appointment was a telephone or video appointment (one patient did not have a view on this question).

### Patient survey comments

Most additional comments centred on the positive experience that people had of feeling listened to, understood and of feeling that care was being coordinated well between different teams. Selected comments from different patients include:

‘More holistic, more attentive’

‘Perfect having several people there, cross-referencing problems’

‘More time, comprehensive’

‘Better balance of medication, all considered at once’

‘Could see there was joined-up thinking’

‘It was calm and people took turns in contributing’

For the three patients who felt that all their problems were addressed ‘somewhat’, further comments about this included: ‘still feels like neck pain is main problem though reassured heart is being checked’, ‘still feel shortness of breath on exertion’.

We asked for feedback on the experience of seeing all the clinicians at the same time in one room and nine patients found this a positive experience that was not intimidating or overwhelming, with a further two patients commenting that they felt nervous when they first entering the clinic room, but not thereafter. When asked how the clinic could be improved, nine patients made no suggestions, but one had not found it easy to understand the letter that they received and would have preferred even simpler written information that they could understand better.

### GP survey

We obtained feedback from two primary care physicians who cared for some of the patients seen in the multispecialty clinic; one had also attended the clinic with their patient. Both agreed that the multispecialty clinic format was different from that of other clinics their patients attended and that it carried ‘more of an MDT’ weight. Both felt that the clinic had reduced the number of other specialty referrals that they would have made for their patients who were seen in the clinic and both felt that the clinic had the correct mix of specialties represented. There was consensus that the clinic review was useful in determining the optimal combination of medications for the patients seen. Both felt that the medical problems were successfully addressed in clinic ‘very well’ and both would like the clinic to be more broadly available and continued in the long term. The GPs were both ‘very satisfied’ with the assessment of the patient in the multispecialty clinic and would recommend the clinic to other GPs.

While neither GP expected all patients to have follow-up visits to the clinic, they were keen that there was a quick and simple way to seek further clarification or discussion of the patients’ ongoing problems if required. Both preferred electronic communication by email in the absence of any direct method of communication through the currently separated primary and secondary care electronic health records. A specific email address for communication after the clinic was suggested. To improve the clinic in the future, it was suggested that letter formatting could include a table of recommendations and a clear and greater emphasis on lifestyle interventions.

### Clinic clinician survey

We interviewed seven clinicians (five consultants, one trainee, one pharmacist) who had worked in the multispecialty clinic, and all felt that the clinic experience was different from that of other clinics they worked in. This was in complete agreement with the responses of patients when they had been asked the equivalent question. All found working in the multispecialty clinic to be a very positive experience and felt that the clinic benefited their professional development ‘very much’ (5) or ‘somewhat’ (2). The clinicians were of the opinion that their professional input was integrated into the overall clinic assessment ‘very well’ (5) and ‘well’ (2). All felt more confident in prescribing decisions for people with MLTC in the clinic with immediate colleague discussion and feedback compared to prescribing in single-specialty clinics, and all felt that patients benefited greatly from being seen in the clinic. All felt that the clinic format improved their ability to help patients’ understanding of their conditions ‘very much’ (3) and ‘somewhat’ (4).

## Discussion

We report our experience of a multispecialty clinic in which people with MLTC met multiple specialist clinicians together in one room and an integrated consensus plan was developed collaboratively. Our aim was to streamline the care of people who might otherwise be attending multiple clinics over time and be receiving multiple specialist opinions that each addressed only the elements of their overall health and experience relevant to that single specialty.

We recognised the potential benefits of this approach, but also the potential challenges, including how people would respond to seeing multiple clinicians at once, how clinicians would operate in this novel situation and how a consensus opinion would be developed and communicated. Therefore, as part of our service evaluation, we elicited feedback from patients attending the clinic, from their primary care physicians and from clinicians conducting the clinic. Our analysis of the clinic, including this feedback, should provide useful insights for the development of patient-centric and better-coordinated services for people living with MLTC.

People were referred to the clinic by clinicians, most commonly from primary care, but also from other services including our same-day emergency care unit. An alternative approach would be to systematically identify people using diagnostic information from their electronic health record. This approach is routinely used for the care of people with diabetes, where blood tests such as HbA1c can be used to identify people who may benefit from further input by primary or secondary care as appropriate.

However, for our clinic, most referrals arose because of a current problem, notably fluid overload, shortness of breath, problems with raised blood pressure or with high glucose levels in diabetes. Although these might be considered common or straightforward issues to manage in the context of a single disease, in the context of MLTC, whether in primary or in secondary care, they can be complex and raise challenging dilemmas. A particular issue can arise where one specialty or even a generalist may be reticent to make a change that impinges on the management of another condition for which the patient sees a different team. We recognise that the majority of day-to-day care for patients with MLTC is performed in primary care and we seek to support this. Most of our referrals were from primary care and so we were responding generally to requests for advice and recommendations that had been solicited by the patients’ GPs.

As anticipated, most people had diabetes, heart failure and CKD – a triad of conditions that, in a study assigning comorbidities to 25 clusters, resulted in the highest number of GP visits and emergency attendances.^23^ In a study of MLTC clusters and adverse outcomes among 1.8 million patients, the largest cluster for adverse outcomes in the 37–54 year age group was hypertension, diabetes and heart failure.^24^ Mental health and pain issues were most prominent in the youngest age group of 18–36 year olds.^24^ However, the presence of physical MLTC is associated with a higher risk of mental health problems.^25^ Since mental health problems are increased in people with MLTC, our clinic team included a consultant psychiatrist participating.^26^ The high prevalence of mental health-related medication usage in our patients highlights the importance of addressing psychological factors in the context of multiple long-term conditions affecting physical health. Attendees of our clinic had a wide age range consistent with studies of the epidemiology of MLTC, which affect people across the age spectrum.^27^

People attending the clinic were being prescribed high numbers of medications but, given the prevalence of diabetes, heart failure and CKD, the use of SGLT2 inhibitors was relatively low and initiation of these drugs was the commonest medication change in the clinic. The consensus real-time multispecialty agreement on the value of a new prescription may have led to more frequent initiation and earlier initiation of these medications than might have arisen in a single-specialty clinic. While for SGLT-2 inhibitors, this may become less of an issue over time as their prescribing is becoming more routine, nevertheless, similar issues are likely to arise recurrently with newer drugs or their broader application and relevance to multiple conditions, such as GLP-1 agonists or newer mineralocorticoid antagonists.

In the 6 months after the clinic, there were almost no new referrals to additional specialist clinics from primary care. Following review in the multispecialty clinic, the clinicians in the clinic decided that some people would need further care in single-specialty clinics for specific reasons, such as preparation for dialysis or consideration for cardiac valve repair, that could not currently be provided in the multispecialty clinic. A key ethos of the clinic was that it sought not to generate any additional unnecessary burden for primary care services, so where a new condition was identified, appropriate action was taken. In this context, review in the clinic identified new conditions requiring input and these included a new cancer and a new spinal condition.

The overall number of unscheduled attendances to the acute care service was reduced in the 6 months after the clinic (comprising the emergency department, SDEC and inpatient acute general medicine). This is notable against a background of year-on-year increases in unscheduled hospital attendances more generally.^28^ We were flexible about whether people were, or were not, followed up in the clinic and, if so, for how long. Most people were seen on only one occasion for a deep and broad assessment, which generally did not need to be repeated over the coming months. If a particular specialty follow-up was required, then we arranged this in one of our own single-specialty clinics.

Surveys of people with MLTC typically show low satisfaction with single-specialty healthcare provision and highlight a lack of holistic care and perceived poor communication between clinicians.^15^^,^^29^ Patients in our multispecialty clinic reported that it was a very different and positive experience compared to their experience in single-specialty clinics. They appreciated the simultaneous input from multiple specialists and did not find the experience overwhelming. Indeed, they generally reported having the right amount of time in the clinic and felt able to ask questions and get answers in real time to their diverse medical problems.

People with MLTC have greater difficulties in understanding their complex health challenges than people with just a single condition.^30^ A key aim of the clinic was to improve people’s understanding of their health and healthcare through a single unified holistic assessment of their situation and the interrelationship between their MLTC. This contrasts with the partial view that people report receiving from attendance at a range of different single-specialty clinics over time. All those attending our clinic reported an increase in their understanding of their health. Future studies could explore how to maximise the understanding gained from the clinic – for example, by providing a recording of the consultation or further written materials or a follow-up phone call.

The GPs felt that a one-off visit to the clinic was very useful and generally did not expect ongoing follow-up. Their suggestions for improvement largely related to improved approaches to post-clinic communication, such as rapid email communication and clarity in documentation of the agreed management plan, to make these easier for multiple members of the primary care multidisciplinary team to follow and execute. The agreed consensus plan was appreciated by the GPs, who found medication changes understandable and actionable. In one case, the GP attended the appointment with their patient, and this was a positive and constructive experience for all concerned. In our recommended plans, we made it very clear which actions would be undertaken by us and which were recommended for primary care to undertake. Any current investigations that we recommended were arranged and followed up by us – examples might include imaging, echocardiography or specialist blood tests. Changes to medication were generally recommendations made for primary care to undertake.

Caring for people with MLTC has been identified as a factor in ‘GP burnout’ and our clinic provides support for GPs in this important domain.^31^ We did not specifically ask about burnout but, overall, the GPs were very positive about the clinic, finding it both a useful one-stop review point for people with MLTC and commenting that it would reduce the number of specialty referrals they would need to make. The multispecialty clinic addresses each of the four challenges identified in a systematic review of GPs’ perspectives on management of people with MLTC, including fragmentation of healthcare, inadequacy of guidelines, challenges in delivering patient-centred care and barriers to shared decision making.^14^

The clinicians enjoyed participating in the clinic and found the teamwork enjoyable, indicating that people with very complex problems can be managed in a way that is fulfilling, not overly stressful and that could contribute to reducing burnout in secondary care. All the clinicians reported a positive benefit for their own professional development from the clinic and felt that they benefited from seeing the management of conditions that are normally the domain of the other specialties. This cross-specialty learning is inherent to the clinic and the need for broadening of medical knowledge beyond a narrow specialist focus is increasingly recognised as a major priority for the NHS.^2^ The clinicians all felt that they had taken knowledge gained in the clinic back to their own individual clinics and used it there productively. Thus, participating in this type of clinic could improve the documented sense of isolation that clinicians report feeling when looking after people with MLTC.^14^

The clinic format involved the specialists all seeing each patient together in one room. The advantage of this was the development of a consensus opinion with the patient in real time and all the clinicians heard the patient talk directly, rather than hearing an account of their problems relayed by someone else. Clinicians and patients both enjoyed this approach, which relies on matching patients with the appropriate mix of specialty clinicians. There was a potential concern that patients had a lot of information to absorb from one visit, but patients did not report feeling overwhelmed with information.

Future work could explore different ways of organising the clinic appointments, different strategies for communicating information to the patient and primary care team, tackling health inequalities and longer-term outcomes and cost–benefit analyses. The cost of an appointment in a multispecialty clinic will be more than that of a single-specialty clinic, but the overall cost may be lower than that of multiple visits to multiple single-specialty clinics. Multiple visits to multiple clinics generate repetitive costs including more administrative costs, more need for letters, which take up clinical and administrative time, likely redundancy in phlebotomy and other tests, greater need for transport and time off work for patients in employment.

## Conclusions

We established a novel multispecialty clinic whereby multiple consultant specialists in the same room saw people with MLTC and developed a consensus management plan with them in real time. Patient, clinician and GP surveys demonstrated very high satisfaction with this approach. Although challenging to organise and using more in-the-moment physician time than an ordinary specialist clinic, targeting people who can benefit from this approach could improve health understanding, experience and potentially outcomes. It also offers the potential to improve clinician wellbeing and education while alleviating discrepancies in patients’ management plans and optimising future healthcare resource usage.

## CRediT authorship contribution statement

**Michael E Reschen:** Writing – review & editing, Writing – original draft, Resources, Methodology, Investigation, Funding acquisition, Formal analysis, Data curation, Conceptualization. **Jennifer J Rayner:** Writing – review & editing, Data curation. **Gaya Thanabalasingham:** Writing – review & editing. **Alistair Lumb:** Writing – review & editing. **Michael Matheou:** Writing – review & editing. **Sophie McGlen:** Writing – review & editing. **Nayia Petousi:** Writing – review & editing. **Luke Solomons:** Writing – review & editing. **Rustam D Rea:** Writing – review & editing, Conceptualization. **Christopher A O'Callaghan:** Writing – review & editing, Writing – original draft, Funding acquisition, Formal analysis, Data curation, Conceptualization.

## Declaration of competing interest

The authors declare that they have no known competing financial interests or personal relationships that could have appeared to influence the work reported in this paper.

## Appendix Supplementary materials

Image, application 1
