# Supplementary material for: Development and evaluation of an integrated multispecialty clinic for people with multiple long-term conditions
Source: Future Healthc J. 2025 Mar 4;12(2):100235. doi: 10.1016/j.fhj.2025.100235 (PMC12002817; doi:10.1016/j.fhj.2025.100235)
Supplement: Supplementary file 1 [file mmc1.docx]

**Supplementary File**

**Clinic clinician/pharmacist/doctor trainee Survey**

Questions about your experience of working in the clinic:

1. Was the clinic experience different compared to other clinics you have worked in? Yes/no/unsure
2. In what way was it different?
3. Any similar clinics?
4. To what extent did you find it a positive experience working in the multispecialty clinic?

Very positive/somewhat positive/neutral/somewhat negative/very negative

1. What did you find good about the clinic?
2. Any aspects that could improve your experience?
3. To what extent do you feel that working in the multi-specialty clinic benefited your professional development, (e.g., learning from cross-specialty collaboration or networking)?

Very much/somewhat/neutral/not much/not at all

1. Can you share any specific examples or experiences from the clinic that highlight the benefits of cross-specialty collaboration or networking?
2. How well do you think your professional input was integrated into the multi-specialty clinic assessments?

Very well/Well/Neutral/Not well/Not at all

1. Any examples or ways to better incorporate healthcare professionals' expertise?
2. Did you feel more confident making medical recommendations for patients knowing you had your colleague specialist’s agreement there and then?

Yes/no/no difference/not sure

1. To what extent do you think the patients benefited from being seen in the multispecialty clinic compared to usual care?

A lot/somewhat/slightly/not at all

1. To what extent do you think the format of the clinic improved the clinician’s ability to foster greater understanding of patient for their conditions?

Very much/somewhat/slightly/not much/not at all

1. What was interesting or stood out about the clinic that is not covered in the questions above?
2. How could the multispecialty clinic format be improved?

**Patent Survey Questions**

1. Was this clinic different from other clinics you have been to? Yes/No
   1. In what way?
   2. Did you like it?
   3. Did you prefer it this way, prefer being under separate clinicians at separate times, or a mixture of systems?
   4. what was it like seeing several healthcare professionals at once in one room.
2. Overall, how satisfied were you with the care you received in the clinic? *Likert scale* Very satisfied/satisfied/neither satisfied nor dissatisfied/dissatisfied/very dissatisfied
3. Did you feel there was the right mix of specialists available for your problems? Yes/No

If not, then who would you have wanted to be there also?

1. How much did you feel that all your problems were addressed at once by seeing several health professionals at once? Very much/somewhat/neutral/not much/not at all

What kinds of problems were not addressed?

1. How much did you feel listened too?

Very much/somewhat/neutral/not much/not at all

1. Did you feel there was enough time during the appointment to discuss your problems with several healthcare professionals? Yes/No

*How much time would be best?*

1. To what extent did the healthcare professionals communicate well with each other in the clinic? Very well/well/ok/not well/badly

Were there any communication issues?

1. To what extent did going to the clinic help you understand your condition better? Very much/somewhat/neutral/not much/not at all
   1. What things could have been gone over better?
2. Have you seen or spoken to your GP since the clinic about issues discussed in the clinic? YES/NO
   1. If yes then – did your clinic appointment with us help you and your GP to work through your medical issues better?
3. What do you think about having the clinic as a virtual appointment by telephone or using video calling – and would you have access to video calling?
4. Do you have any other thoughts on how the clinic could be improved?

**Primary Care GP survey questions**

1. Overall, how satisfied were you with the assessment of your patient in the multispecialty clinic?

Very satisfied/somewhat satisfied/neutral/somewhat dissatisfied/very dissatisfied

1. Did the assessment of your patient in the multispecialty clinic format seem different to other clinics your patients attend?

Yes/No/Unsure

1. Did utilizing the multispecialty clinic affect the number of other specialty referrals you made or would have made for the patient?

Increased referrals/no change/decreased referrals

1. How useful was the multispecialty clinic assessment in answering queries about the optimal combination of medication?

Very useful/somewhat useful/neutral/not very useful/not useful

1. Was the right mix of specialists available for your patient?

Yes/No If no, which other specialties would it have been useful to include?

1. To what extent were your patients’ medical problems successfully addressed by the clinic?

Very well/well/neutral/not very much/not at all

1. Would you consider it useful if the clinic was more broadly available and continued as a long-term entity?

Yes/No/Unsure

1. Were you satisfied with the level of coordination and communication prior to your patient's appointment?

Yes/No/Unsure

1. Were you satisfied with the level of coordination and communication between the different specialists involved after your patient's appointment?

Yes/No/Unsure

1. How well did the integrated multi-morbidity clinic meet the healthcare needs of your patient? Greatly, somewhat, neutral, not very much, not at all
2. Would you recommend this pilot multi-morbidity clinic to other GPs?

Yes/No/Unsure

1. Did the review in the integrated multimorbidity clinic make it clearer how to best follow-up the patient’s medical problems?

Very much/somewhat/neutral/not very much/not at all

1. Would you prefer your patients to have either:
   1. ongoing follow-up appointments in the multimorbidity clinic
   2. one-off appointment with the option to re-refer as required
   3. unsure
2. Would you like to participate during the actual appointment your patient has (in person, telephone or video link)?

Yes/No/Unsure

1. How practical was it to follow the advice from the multimorbidity clinic? Slightly negative question

Easy/ normal / difficult?

1. How could the multispecialty clinic help make it easier to implement the suggested recommendations for care?
2. Would it be useful to have access to a direct communication with a clinician after the appointment either by email or telephone over or otherwise and above existing methods? Yes/No/Unsure.
3. What aspects of the integrated multi-morbidity clinic do you believe could be improved to better serve patients and referring GPs?
